# Supplementary material for: Electrically activated ferroelectric nematic microrobots
Source: Nat Commun. 2024 Aug 20;15:6928. doi: 10.1038/s41467-024-50226-y (PMC11336208; doi:10.1038/s41467-024-50226-y)
Supplement: Supplementary file 2 — Description of Additional Supplementary Files [file 41467_2024_50226_MOESM2_ESM.docx]

**Description of Additional Supplementary Files**

**File Name: Supplementary Video 1
Description:** Dynamic breakup regime.

**File Name: Supplementary Video 2
Description:** Moving febots with 750 nm thick insulating layers.

**File Name: Supplementary Video 3
Description:** Moving febot with 1.5 um thick insulating layers.

**File Name: Supplementary Video 4
Description:** Moving febots with 3um thick insulating layers.

**File Name: Supplementary Video 5
Description:** Moving febots on teflon layers.

**File Name: Supplementary Video 6
Description:** Febots for tracking with circular.

**File Name: Supplementary Video 7
Description:** Febots recorded at 60000 fps.

**File Name: Supplementary Video 8
Description:** Febots recorded at 60000 fps – every 1000th frame is shown.
